# The role of non-hematopoietic stromal cells in the persistence of inflammation

**DOI:** 10.3389/fimmu.2012.00416

**Published:** 2013-01-14

**Authors:** Francesca Barone, Saba Nayar, Chris D. Buckley

**Affiliations:** Centre for Translational Inflammation Research, Arthritis Research UK, Rheumatology Research Group, School of Immunity and Infection, College of Medical and Dental Sciences, University of Birmingham Research Laboratories, Queen Elizabeth HospitalBirmingham, UK

**Keywords:** endothelium, lymphatics, rheumatoid arthritis, ectopic lymphoneogenesis, fibroblasts

## Abstract

Inflammation results from the complex interaction between hematopoietic and stromal cells and growing evidence supports a key role for the stroma in driving the switch from acute resolving to persistence in chronic inflammatory diseases. Stromal cells have also been shown to play a critical role in cancer biology, being involved in cancer growth, dissemination, and inhibition of the autologous immune response, ultimately favoring persistence and metastatic spread. Similarly, blood and lymphatic endothelial cells contribute to tissue homeostasis during physiological inflammation but also lead to discorded leukocyte and tumor cell accumulation in pathological inflammation and cancer. This review aims to summarize the role that pathogenic stroma plays in the pathogenesis of diseases such as cancer and chronic inflammation.

## INTRODUCTION

Inflammation involves the complex interaction between infiltrating cells, belonging to the immune system and tissue-resident stromal cells. The stroma, formerly considered the theatrical stage of the inflammatory process, has acquired, in recent years the role of director of the immune response, regulating the process of leukocyte recruitment, organization of leukocytes within the tissue and exit via the escape route of the lymphatic endothelium.

In physiological conditions, stromal cells provide an important structural component for tissues. Stroma consists of extracellular matrix (ECM), mesenchymal cells and a scaffold of nerves, epithelium, blood, and lymphatic vessels. Tissue-resident macrophages, although considered by some as part of the tissue stroma, are largely bone marrow-derived and bear the hallmark of the hematopoietic tissue.

Under pathogen threat or in the target tissue of chronic inflammation and cancer, stromal cells acquire novel features, critical for the development of the pathological process and functional for its persistence. This review will provide the reader with a better understanding of the role of pathogenic stroma in inflammation. As both epithelium and nerves appear to be more implicated in disease initiation, rather than persistence, this review will focus on the role of fibroblasts, lymphatic and blood vessels.

## FIBROBLASTS

The most abundant cell type within tissue stroma is the fibroblast (Filer et al., 2006). Fibroblasts are traditionally defined by their spindle shaped morphology and their ability to adhere to plastic culture vessels *in vitro* (Tarin and Croft, 1969). They are believed to arise from three distinct cellular origins: primary mesenchyme, local epithelial-mesenchymal transition (EMT), or bone marrow-derived precursors (circulating fibrocytes; Abe et al., 2001; Kalluri and Neilson, 2003). It is widely accepted that the majority of fibroblasts originate from primary mesenchymal cells and that, upon appropriate stimulation, fibroblasts proliferate to generate new progeny (Iwano et al., 2002; Parsonage et al., 2005). In physiological conditions fibroblasts provide mechanical strength to tissues by producing ECM components (type I, III, and V collagen and fibronectin), factors that regulate ECM turnover, such as metalloproteinases (MMPs) and proteins involved in the formation of basement membrane (type IV collagen and laminin; Marinkovich et al., 1993; Sabatelli et al., 2001; Tomita et al., 2002). The intimate relationship between fibroblasts and mesenchymal stromal cells (MSC) and the clinical challenge to use MSC for tissue repair has driven renewed interest in fibroblasts as therapeutic target. Our group has largely contributed to this characterization utilizing antibodies raised against different components of this heterogeneous population. This screening exercise has provided us with some key markers that, together with others present in the literature (**Table 1**), can now be used to better understand distribution, function, and plasticity of the complex fibroblast family (Buckley et al., 2005; Halder et al., 2005).

**Table 1:** Fibroblast markers.

Marker	Function	Fibroblast subtype	Other expressing cells
Vimentin	Intermediate filament associated protein	Miscellaneous	Endothelial cells, myoepothelial cells, neurons
α-SMA	Intermediate filament associated protein	Myofibroblasts	Vascular smooth muscle cells, pericytes, myoepithelial cells
Desmin	Intermediate filament associated protein	Skin fibroblasts	Muscle cells, vascular smooth muscle cells
FSP1	Intermediate filament associated protein	Miscellaneous	Invasive carcinoma cells
Discoidin-domain receptor 2	Collagen receptor	Cardiac fibroblasts	Endothelial cells
FAP	Serine protease	Activated fibroblasts	Activated melanocytes
α1β1 integrin	Collagen receptor	Miscellaneous	Monocytes, endothelial cells
Prolyl 4-hydroxylase	Collagen biosynthesis	Miscellaneous	Endothelial cells, cancer cells, epithelial cells
Pro-collagen 1α2	Collagen-1 biosysnthesis	Miscellaneous	Osteoblasts, chondroblasts
CD248	Unknown	Miscellaneous	Pericytes
VCAM-1	Cell adhesion	Miscellaneous	Activated endothelial cells

Adapted from Kalluri and Zeisberg (2006).

Fibroblast behavior has mainly been explored in three pathological conditions: chronic inflammation, tissue fibrosis, and cancer. Interestingly, while these three conditions dramatically differ in etiology and genetic predispositions, they converge in that there are profound modifications both in terms of phenotype and function occurring to the stromal component. Whether these newly acquired properties are intrinsic to changes occurring in the fibroblasts or derive from the conditioning of the pathogenic infiltrating cells is still under investigation and seems to differ in the diverse conditions (Buckley et al., 2001).

Fibroblasts play a crucial role in determining the site at which inflammation occurs, and influence the persistence of the inflammatory process (Takemura et al., 2001). Different events have been shown to take place in order to elicit the modifications required for fibroblast activation. Signals derived from the surrounding infiltrating cells, such as proinflammatory cytokines have been shown to play a key role in the activation of rheumatoid arthritis (RA) synovial fibroblasts (Ohata et al., 2005). Similarly, leukocyte-derived signals such as IL-4 (Th2), interferon gamma (Th1), and TNF have been shown to modify the fibroblast transcriptional profile (Parsonage et al., 2003). Nonetheless, a growing body of evidence suggests that intrinsic events such as the occurrence of epigenetic modifications in the fibroblast genome might contribute to the persistence of the activated phenotype (Ospelt et al., 2011).

Once activated, synovial fibroblasts have been shown to produce TNFα, IL-1, and IL-6, cyclooxygenase-2, the polysaccharide hyaluronan, as well as inflammatory chemokines (e.g., IL-8, CCL5, CXCL1; Szekanecz et al., 2003; Iwamoto et al., 2008), thus sustaining leukocyte recruitment in to the inflamed synovium. Fibroblasts play not only a key role in immune cell recruitment but also in leukocyte aggregation within tissue. Pathogenic fibroblasts have been implicated in the formation of tertiary lymphoid organs (TLOs), lymph node like structures, resulting from the organized aggregation of leukocytes inside tissue target of inflammatory processes (Buckley et al., 2000). Recent work of Peduto et al. (2009) has described early modifications occurring to normal stromal fibroblasts following inflammatory stimuli, like the re-expression of the fibroblast embryonic marker gp38, that result in the acquisition of a lymphoid like phenotype able to sustain TLO formation. Gp38 is a glycoprotein characterized by Farr et al. (1992), later named podoplanin due to its low level constitutive expression in kidney podocytes (Breiteneder-Geleff et al., 1997). Gp38 (or podoplanin in humans) is expressed by lymphoid stromal cells within the T cell areas of peripheral lymphoid tissue (Farr et al., 1992), in the medulla and paracortex of lymph nodes, within the peri-arteriolar region of the splenic white pulp (PALS), on the lymphatic endothelial cells (Schacht et al., 2003) and on thymic epithelial cells (Farr et al., 1992). The role of gp38 + fibroblasts in the production of lymphoid cytokines and chemokines in secondary lymphoid organs has been reviewed elsewhere and will not be discussed further in this review (Astarita et al., 2012).

In physiological conditions, within non-lymphoid tissue, fibroblasts do not express gp38. Interestingly, the phenomenon of up-regulation of this marker coincides with the capacity of tissue-resident fibroblasts to “convert to a lymphoid-like” functional phenotype. Lymphoid-like fibroblasts express CD157 (BP-3) and produce IL-7 and lymphoid chemokines CXCL13 and CCL19 which are able to drive accumulation and segregation of the leukocytes in distinct compartments within the inflamed joints (Buckley et al., 2000, 2001; Bradfield et al., 2003; Peduto et al., 2009). The histological finding of TLOs in RA synovium has been associated with severe disease progression and erosions (van de Sande et al., 2011). TLOs are not specific to RA and other chronic diseases, such as Sjogren’s syndrome, Hashimoto thyroiditis, and Crohn’s disease share a similar pattern of fibroblast activation and production of lymphoid cytokines/chemokines (Aloisi and Pujol-Borrell, 2006).

Rheumatoid arthritis synovial fibroblasts produce survival factors (e.g., type I interferon, IL-15, BAFF) that inhibit leukocyte apoptosis (Pilling et al., 1999; Burger et al., 2001). Gp38 expression is associated with the acquisition of a motile, contractile phenotype and it has been detected in cells derived from various types of cancers (i.e., vascular tumors, tumors of the central nervous system, malignant mesothelioma, squamous cell carcinomas, and germ cell tumors). Gp38 expression seems to identify more aggressive forms of tumors, with higher invasive and metastatic potential (Schacht et al., 2005; Raica et al., 2008). Gp38 is expressed both by tumor cells and by the cancer-associated fibroblasts (CAF), a population of fibroblasts that surrounds and mingle with the malignancy favoring its organization and metastasis in to the surrounding tissue. The expression of gp38 in the context of tumor-associated lymphangiogenesis will be later discussed. CAF as well as fibroblasts from the inflamed synovium are also characterized by FAP (fibroblast activation protein) expression (Ospelt et al., 2010).

Fibroblast activation protein, also known as “seprase,” is a cell-surface 170 kDa type II transmembrane serine protease (Rettig et al., 1986; Aoyama and Chen, 1990), belonging to the family of post-prolyl aminopeptidases (Niedermeyer et al., 1998). Dipeptidyl peptidase IV (DPPIV or CD26) is the most studied closest member to FAP, with 61% nucleotide sequence and 48% amino acid sequence identity (Scanlan et al., 1994). FAP was identified as an inducible antigen by F19 monoclonal antibody and expressed on developing (Rettig et al., 1988; Garin-Chesa et al., 1990; Niedermeyer et al., 2001) and reactive mesenchyme of various tumors, transformed cell lines, and granulation tissue of wound healing (Rettig et al., 1986, 1988; Aoyama and Chen, 1990; Garin-Chesa et al., 1990; Kelly et al., 1994; Monsky et al., 1994). When over-expressed in epithelial and fibroblastic cell lines, FAP has been proven to affect cell adhesion, migration, proliferation, and apoptosis (Wang et al., 2005).

Recently a novel immunosuppressive role for FAP-positive fibroblasts has been shown in the tumor environment. By using a FAP-DTR mice, in which deletion of FAP + fibroblasts is induced upon diphteria toxin administration, Kraman et al. (2010) have shown that depletion of FAP-expressing cells in Lewis lung carcinoma and pancreatic ductal adenocarcinoma causes rapid hypoxic necrosis of both tumor and stromal cells by a process involving IFNγ and TNFα. These studies support the hypothesis that FAP activity and FAP-expressing fibroblasts facilitate tumor growth both directly as well as acting on the immune cells recruited against the malignancy. This suggests that cancerous cells, early in the disease establishment are able to modify the local environment and induce the formation of a stroma able to protect the same malignancy against the self-immune-surveillance, thus establishing a novel immunological role for stromal cells in cancer persistence and spreading.

## VASCULAR STRUCTURES

### LYMPHATIC VESSELS

Striking changes in the lymphatic vasculature are associated with inflammation, which include acute and chronic infections, autoimmune diseases such as RA, Crohn’s disease, wound healing, cancer, and transplant rejection (Tammela and Alitalo, 2010; Alitalo, 2011). Neo-lymphangiogenesis is a critical mechanism regulating changes in interstitial fluid. Deregulated activation of its cascade results in defective leukocyte drainage and persistence of the inflammatory process. Recent studies show that induction of the NF-κB pathway activates Prox1 and this in turn activates the expression of the VEGFR-3 promoter, leading to increased receptor expression on lymphatic endothelial cells. This phenomenon enhances the responsiveness of pre-existing lymphatic endothelium to VEGFR-3 ligands, VEGF-C and VEGF-D, which stimulates lymphangiogenesis (Alitalo et al., 2005; Zhang et al., 2007; Watari et al., 2008; Kang et al., 2009; Flister et al., 2010). Other proinflammatory cytokines, e.g., IL-1 and TNFα are known to induce VEGF-C/D expression in infiltrating and tissue-resident cells such as macrophages, dendritic cells (DCs), mast cells, and fibroblasts (Ristimaki et al., 1998; Hamrah et al., 2003; Cursiefen et al., 2004; Alitalo et al., 2005; Baluk et al., 2005; Kataru et al., 2009; Kunder et al., 2009, 2011; Yao et al., 2010; Zumsteg and Christofori, 2012). Similarly, LTα secreted by lymphocytes at the site of inflammation has been documented to support inflammatory lymphangiogenesis (Mounzer et al., 2010). Data from models of inflamed cornea in mice and renal transplant induced inflammation in humans have shown that inflammation-mediated lymphangiogenesis does not occur solely by proliferation or continuous sprouting of existing lymphatic vessels but also includes incorporation of BM-derived lymphangiogenic progenitors (such as CD11b + macrophages) into the existing or growing lymphatic vessels. These CD11b + progenitors have the capability to trans-differentiate into LYVE + vessels under pathological conditions, contributing to the increased lymphatic vessel density observed at inflammatory sites (Maruyama et al., 2005; Kerjaschki et al., 2006; Maby-El Hajjami and Petrova, 2008; Lee et al., 2010). Lymphangiogenesis is thought to facilitate resolution of inflammation, providing drainage of tissue edema, clearance of inflammatory cells and also favoring adaptive immune cell function by promoting macrophage and DCs mobilization to the draining lymph nodes. Inhibiting lymphangiogenesis by blocking VEGFR activity exacerbates pulmonary edema and favors the establishment of chronic mycoplasma infection (Baluk et al., 2005; Polzer et al., 2008; Kataru et al., 2009; Huggenberger et al., 2011; Kim et al., 2012). Taken together, these data suggest that the signals that activate the induction of the inflammatory process also program its resolution. It is believed that such a coordinated series of events is altered in chronic inflammation, where the number of the infiltrating leukocytes overcome the drainage capacity of the newly developing lymphatics. It is debatable whether newly formed lymphatic vessels are able to establish functional connections and deliver antigen, fluid, and cells to the draining lymph nodes. The inability of lymphatic vessels to deliver antigens to the draining lymph node could favor the persistence of TLOs, where an excess of antigen is presented in structures whose stroma component is potentially unable to exert the tollerogenic activity attributed to lymph node stroma (Kline and Thomas, 1976; Eliska et al., 1986; Joos et al., 1993; Ruggiero et al., 1994; Angeli et al., 2006; Angeli and Randolph, 2006; Thaunat et al., 2006; Li et al., 2011). In this context the ectopic expression of CCL21 on newly formed (but yet non-functional lymphatic vessels) might compete with the chemokine gradient established by pre-existent functional lymphatic vessels, thus contributing to the entrapment of leukocytes and DCs within the TLOs (Kerjaschki et al., 2004; Burman et al., 2005).

Lymphatic vessels have also been shown to play a role in immune regulation. For example the decoy chemokine receptor D6, which acts as a scavenger of inflammatory CC chemokines, is known to be expressed on lymphatic endothelium (Nibbs et al., 2001; Jamieson et al., 2005; Martinez de la Torre et al., 2005). In addition, inflamed lymphatic endothelium mediates suppression of DC maturation via CD11b interaction with ICAM-1 receptor (expressed on lymphatic endothelial cells; Podgrabinska et al., 2009).

However, lymphangiogenesis is a double-edged sword, as tumor cells can use this process as a mechanism to drive metastasis. Tumor cells enter lymphatic vasculature by invading pre-existing lymphatic vessels or by eliciting neo-lymphangiogenesis on the periphery of the tumor, stimulating the secretion of growth factor (VEGF) on tumor-associated fibroblasts and macrophages (Jeltsch et al., 1997; Karpanen et al., 2001; Mandriota et al., 2001; Skobe et al., 2001; Stacker et al., 2001; Schoppmann et al., 2002). Interestingly, high density of lymphatic vessels correlates with high incidence of lymph node metastasis and poor prognosis in some cancers (Beasley et al., 2002; Alitalo et al., 2005; Cao, 2005; Kyzas et al., 2005). As mentioned above, gp38 is highly expressed on the lymphatic endothelium and its expression correlates with poor prognosis and increased risk of lymph node metastasis (Schacht et al., 2005; Kitano et al., 2010). Lymphangiogenesis has also been found to play a pathogenic role in transplantation biology where it sustains delivery of donor antigens to the recipient lymph node, ultimately favoring the generation of an immune response against the transplanted tissue (Tammela and Alitalo, 2010; Alitalo, 2011; Seeger et al., 2012). These data suggest a key role for lymphatic vessel function and homeostasis in the regulation of the balance between immunity and tolerance as well the persistence of inflammation compared to its resolution.

### BLOOD VESSELS

Similar to the fibroblasts and lymphatic vessels, blood vessels undergo remodeling during inflammation. Blood endothelium changes its structure and phenotype and participates in the inflammatory response mainly regulating leukocyte recruitment into the tissue. This phenomenon is characterized by loss of vascular integrity, which results in exposure of the sub-endothelium matrix and efflux of plasma-protein rich from the intravascular space (Pober and Sessa, 2007). The newly formed extravascular matrix supports leukocyte extravasations and it is associated with the expression of leukocyte adhesion molecules such as E-selectin, VCAM-1, and ICAM-1 (Adams and Shaw, 1994; Clark et al., 2006; Ley and Reutershan, 2006). Inflammatory stimuli such TNFα, IL-1, certain bacteria and viruses, physical and oxidative stress (Pober and Sessa, 2007), and anti-endothelial cell antibodies (found in systemic inflammatory disease such as vasculitides; Meroni et al., 1995) all elicit NF-κB translocation and binding to promoter regions of genes commonly up-regulated during blood endothelial cell activation (Bierhaus et al., 1997; Hunt and Jurd, 1998; Pober and Sessa, 2007). Failure to restore homeostasis of the blood endothelium contributes to chronic inflammatory disease and edema. Activated blood endothelial cells synthesize cytokine such as IL-6, which regulates the acute phase response, and chemoattractants, such as IL-8 and MCP-1 that help establishing the chemotactic gradient necessary for the influx of various inflammatory cells into the site of inflammation (Pober and Cotran, 1990; Mantovani et al., 1997; Middleton et al., 1997). Blood endothelial cells are also able to act as an antigen-presenting cell, expressing class II HLA molecules, in a phenomenon that has been shown to contribute to transplant rejection (Pober et al., 1996). Expression of co-stimulatory molecules such as OX40, ICOS, and CD2, known to be important in the formation and activation of T cell memory, has been documented in activated human endothelial vessels (Shiao et al., 2005), suggesting a role for the endothelium not only in leukocyte recruitment but also in their education.

Additional endothelial changes are observed in various chronic inflammatory diseases, such as Sjogren’s syndrome, thyroiditis, and RA. As mentioned above, chronically inflamed organs often acquire TLOs that are accompanied by conversion of flat venular endothelial cells into tall and plump endothelial cells that very closely resemble high endothelial venules (HEVs) found in the T cell rich area of the lymph node. These ectopic HEVs are characterized by expression of the lymph node trafficking code, Peripheral node addressin (PNAd) that binds L-selectin expressed on naïve/central memory T lymphocytes and mature DCs. This homing machinery, supported by the ectopic expression of CCL21, allows HEVs in peripheral tissue to misguide influx of CCR7 + memory T cells into the inflamed tissue leading to amplification and maintenance of chronic inflammation (Girard and Springer, 1995; Barone et al., 2005; Manzo et al., 2005; Aloisi and Pujol-Borrell, 2006). Our group has recently demonstrated that endothelium cultured with fibroblasts derived from inflamed synovium behaves differently in terms of leukocyte recruitment compared to endothelium cultured with healthy dermal fibroblasts (McGettrick et al., 2009). While reinforcing the concept that fibroblasts convey site specificity to the immune response, these data highlight the active role of the stroma in the shaping of the immune response from its earliest phases.

Growth of new blood vessels from existing ones is a very important component of many diseases including cancer and chronic inflammation (Pandya et al., 2006; Costa et al., 2007). Angiogenesis in these conditions ensures continuous oxygen and nutrient supply to pathogenic cells, thus sustaining their growth and survival. Several cells types including malignant cells, synovial fibroblasts, keratinocytes, and monocytes/macrophages are capable of producing classic angiogenic factors (such as VEGFα, angiopoetin, and PDGF) when the environment becomes hypoxic. Moreover, inflammatory cytokines such as IL-1, TNFα (low dose), and IL-8 have been reported to be pro-angiogenetic, thus supporting this process while exerting other proinflammatory activity. Blood vessels formed during pathological angiogenesis are structurally and functionally abnormal. Tumor vasculature is highly disorganized, vessels are tortuous and dilated, with uneven diameter, excessive branching and shunts that lead to chaotic and variable blood flow, often resulting in the establishment of hypoxic and acidic areas in the malignant tissue. The chaotic architecture and disturbed blood flow contributes to lower therapeutic effectiveness for several drugs (Jain, 1988; Giaccia, 1996; Helmlinger et al., 1997; Baish and Jain, 2000; Eberhard et al., 2000). Malignancy-associated neo-vascularization shows a non-uniform pattern of adhesion molecules that coupled with distorted blood supply (Huang et al., 1997; Eliceiri and Cheresh, 1999) might explain why leukocyte-endothelial interaction is low in cancer. Similarly, the new vessels formed at the site of inflammation also exhibit structural and functional abnormalities. In RA, the vascular network is reported to be dysfunctional as it is unable to restore tissue oxygen homeostasis and the rheumatoid joints are believed to remains markedly hypoxic during disease (Taylor and Sivakumar, 2005). In both tumor and RA impaired angiogenesis ultimately favors the selection of cells that are metabolically resistant to lack of oxygen, thus reducing the effectiveness of therapy aimed to disturb the neo-angiogenetic process.

## CONCLUSION

Current treatments targeting leukocytes have led to a dramatic change in the management of inflammatory disease. However, this approach does not result in a definitive cure and even in patients that achieve clinical remission, relapse occurs once treatment is withdrawn. This suggests that other non-leukocyte targets need to be addressed if persistent inflation is to be resolved. In this review we have explored the role of stromal cells in disease pathogenesis and suggest that stromal cells provide an attractive, site-specific target for therapy. Combined therapies, aimed at silencing the stroma, provide an alternative approach to curing persistent inflammatory disease.

## Conflict of Interest Statement

The authors declare that the research was conducted in the absence of any commercial or financial relationships that could be construed as a potential conflict of interest
